# Predictors of Early and Long‐Term Sound Quality Ratings in Adult Cochlear Implant Recipients

**DOI:** 10.1002/lary.70007

**Published:** 2025-08-22

**Authors:** Katelyn A. Berg, Terrin N. Tamati, Aaron C. Moberly

**Affiliations:** ^1^ Otolaryngology—Head and Neck Surgery Vanderbilt University Medical Center Nashville Tennessee USA

**Keywords:** cochlear implant, patient‐reported outcomes, quality of life, sound quality, SSQ‐12

## Abstract

**Objective(s):**

To characterize the trajectory of subjective sound quality perception over time in adult cochlear implant (CI) recipients and identify predictors of early and long‐term sound quality outcomes.

**Methods:**

In this retrospective analysis, 339 adult CI recipients completed the speech, spatial and qualities of hearing scale (SSQ‐12) pre‐operatively and at 1, 6, and 12 months post‐activation. Demographic, audiometric, and electrode placement data were analyzed using linear mixed effects models to identify predictors of sound quality ratings on the SSQ‐12 qualities subscale.

**Results:**

Sound quality ratings showed statistically significant improvement from pre‐operative baseline to 1‐month and from 1‐ to 6‐months post‐activation, with no significant change between 6‐ and 12‐months. Better early (1‐month) sound quality ratings were significantly predicted by better pre‐operative sound quality ratings, better word recognition, closer modiolar distance, and a deeper insertion depth. Better long‐term (6–12 month) sound quality ratings were significantly predicted by younger age, greater daily CI processor use, and better 1‐month sound quality ratings.

**Conclusion:**

Perceived sound quality in CI recipients plateaued by 6 months post‐activation. While electrode placement factors and better pre‐op word recognition significantly predicted early sound quality outcomes, younger age and greater device use predicted better long‐term sound quality ratings, highlighting opportunities for targeted intervention to optimize sound quality outcomes. Importantly, these findings underscore the need for more sensitive sound quality assessment tools to better evaluate surgical and patient factors that affect recipients' subjective auditory experiences.

**Level of Evidence:**

4

## Graphical abstract

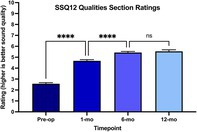

## 1 Introduction

For the 430 million people worldwide with moderate to profound hearing loss, cochlear implants (CIs) are the standard treatment to restore functional hearing [1, 2]. However, CI outcomes vary significantly, with sound quality experiences ranging from perceiving mostly white noise to achieving near‐natural hearing [3]. In recent years, research emphasis has broadened beyond speech recognition toward measuring more patient‐centered outcomes, such as CI‐related quality of life (CI‐QOL) [4]. Importantly, speech recognition and quality of life outcomes have been found to be largely independent [5], suggesting that traditional speech recognition metrics may not capture all aspects of hearing that matter to recipients. Instead, subjective sound quality as experienced in everyday life—encompassing multiple dimensions such as clarity, naturalness, resonance, and distortion—has emerged as a potential strong predictor of CI‐QOL outcomes. In fact, subjective sound quality has been shown to explain up to 50% of variability on a measure of CI‐QOL, the Cochlear Implant Quality of Life (CIQOL‐35) instrument [6]. This finding stands in stark contrast to demographic and audiometric predictors, which maximally explain only 8%–17% of the variance in CIQOL‐35 outcomes [7]. These findings suggest that perceived sound quality may be a critical but understudied indicator of hearing‐related quality of life and patient satisfaction.

The speech, spatial and qualities (SSQ‐12) questionnaire provides a validated clinical tool for assessing subjective aspects of hearing, including sound quality using the qualities subscale [8]. As part of the minimum speech test battery‐3 [9], the SSQ‐12 qualities subscale specifically assesses four key aspects of sound quality: the perception of multiple sounds as distinct rather than jumbled, the ability to identify different musical instruments, the clarity of everyday sounds, and the level of concentration required when listening. These items capture dimensions of the real‐world CI listening experience that extend beyond understanding speech in the controlled conditions of the sound booth.

While it is well‐documented that adult CI recipients typically demonstrate rapid gains in speech recognition during the first 6 months followed by a plateau after 6–12 months [10, 11], much less is known about how sound quality evolves over time. Dorman and colleagues provided some insight by asking adult CI recipients to retrospectively describe their sound quality experiences around the time of CI activation, with terms like “computer‐like,” “treble‐y,” “metallic,” and “Mickey Mouse‐like” being most common [12]. When describing their current sound quality 4–8 years post‐activation, “clear” emerged as the predominant descriptor. However, that retrospective approach was limited by recall bias. The longitudinal trajectory of sound quality perception, particularly in the first 12 months post‐activation, remains poorly characterized.

This critical knowledge gap regarding the evolution of sound quality perception post‐CI activation limits our ability to properly counsel patients about what to expect regarding sound quality and to develop interventions to improve sound quality outcomes. The present study addresses these limitations through a longitudinal analysis of sound quality ratings, measured via the qualities subscale of the SSQ‐12, in a large cohort of adult CI recipients. Our objectives were to: (1) characterize the trajectory of sound quality ratings from the pre‐operative evaluation through 12 months post‐activation, and (2) identify demographic, electrode placement, and audiometric factors that predict early (1‐month) and long‐term (6–12 months) sound quality outcomes. We hypothesized that sound quality ratings would significantly improve following activation but plateau before 1 year post‐activation, consistent with the time course of speech recognition outcomes [10, 11]. Additionally, we hypothesized that better sound quality would be predicted by younger age, greater daily processor use, full scala tympani insertion, better acoustic hearing, and better speech recognition scores [7, 13]. Although speech recognition and sound quality are not strongly correlated [6], we hypothesized that similar demographic and audiometric factors may predict sound quality outcomes since sound quality perception relies on the auditory signal transmitted by the CI as well as any residual acoustic hearing.

## 2 Methods

**FIGURE 1:**
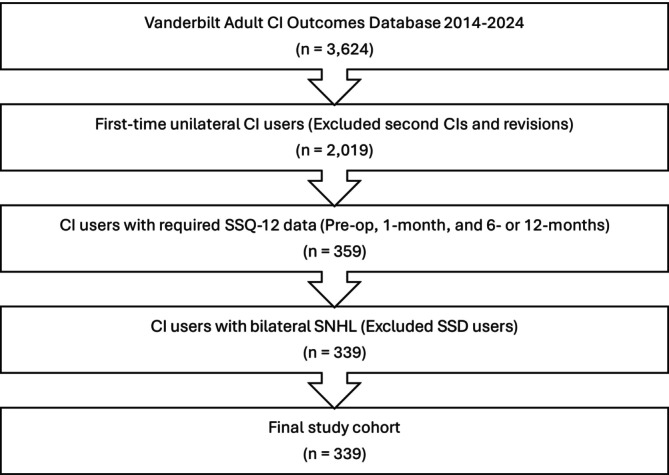
Patient selection flowchart showing exclusion criteria applied to the Vanderbilt adult CI outcomes database (2014–2024). A total of 339 participants with bilateral sensorineural hearing loss and complete SSQ‐12 data were included in the final analysis after excluding participants with second CIs/revisions, incomplete data, and single‐sided deafness.

Exemption was obtained for this study from our Institutional Review Board (IRB). A retrospective analysis examined data from adult cochlear implant (CI) recipients followed longitudinally across four timepoints: pre‐op, 1‐month, 6‐months, and 12‐months post‐activation. Data were collected from clinical visits at a single academic medical center between 2014 and 2024, with analysis performed in March 2025. Inclusion criteria required participants to have speech, spatial and qualities of hearing scale (SSQ‐12) qualities sub‐scale data for pre‐op, 1‐month, and either the 6‐ or 12‐month timepoints. Participants were excluded if they had two CIs to focus on the impact of the first intervention of electric hearing on sound quality. They were also excluded if they met single‐sided deafness (SSD) candidacy criteria (pure tone average (PTA) in the implanted ear of >80 dB HL and PTA in the contralateral ear of ≤ 30 dB HL) [14], as SSD users demonstrate substantially different subjective sound quality experiences compared to bilateral sensorineural hearing loss users [12]. Figure 1 displays the patient selection process, resulting in a final study cohort of 339 participants.

The primary outcome measure was sound quality, assessed using the qualities sub‐scale of the SSQ‐12. The SSQ‐12 items are rated on a scale from 0 (complete disability) to 10 (complete ability). Qualities scores are the average of the item ratings across the four questions described in the Introduction. Demographic and audiometric data were collected from the electronic health record, including age at CI, hearing loss onset (pre‐ [< 3 years old] vs. post‐lingual [> 3 years old]), age first fit with hearing aids, CI manufacturer (Advanced Bionics, Cochlear, or MED‐EL), electrode type (precurved or straight), scalar location (full scala tympani [ST] or translocated into scala vestibuli [SV] insertion), average electrode‐to‐modiolus distance in mm across the array, angular insertion depth in degrees [15], ipsilateral (implanted ear) pure‐tone average unaided, ipsilateral low‐frequency (125–500 Hz) pure‐tone average unaided, contralateral (non‐implanted ear) pure‐tone average unaided, contralateral low‐frequency (125–500 Hz) pure‐tone average unaided, and aided consonant‐nucleus‐consonant (CNC) word recognition [16] scores in the ear to be implanted (pre‐op) aided with a hearing aid fit to NAL‐NL2 targets [17] and in the CI alone condition (post‐op). The contralateral ear was either plugged/muffed or masked via insert earphone during CNC word recognition testing. Electrode placement information was determined using computed tomography (CT) imaging with validated CI position analysis algorithms [15].

All analyses were conducted in R version 4.4.1 [18]. Sound quality ratings were grouped into three timepoints: preop (T1), early (1‐month; T2) scores, and long‐term (6–12 months; T3) scores. For participants with both 6‐ and 12‐month scores, the higher of the two scores was used as the T3 score in analyses to capture maximal scores. To address occasional missing data across clinical visits, multiple imputation was employed using the mice package with predictive mean matching, generating 10 imputed datasets with 20 iterations each. Results were pooled across these imputed datasets following Rubin's rules [19] to ensure robust parameter estimates.

To examine the relationships between the variables, a heterogeneous correlation matrix was calculated using a Bonferroni‐corrected alpha level of 0.000417 for the 120 comparisons. Variance inflation factor (VIF) scores were also calculated. Variables that were moderately correlated (*r* ≥ 0.5) or had a VIF score > 10 were excluded from model building [20]. Models prioritized continuous (e.g., modiolar distance) over categorical (e.g., electrode type) variables to maximize statistical power. Based on these criteria, the following variables were excluded from final regression models: age fit with hearing aids (HAs), CI manufacturer, electrode type, ipsilateral low‐frequency PTA, contralateral PTA, and contralateral low‐frequency PTA. Ipsilateral PTA was kept in the models instead of contralateral PTA due to significant missing data at the 1‐ and 6‐month timepoints for contralateral PTA. Extracochlear electrodes were also excluded due to minimal occurrence and range (seven participants with one electrode each), limiting predictive utility.

To examine changes in sound quality over time, a mixed‐effects model was conducted on sound quality ratings across timepoints, with timepoint as a fixed effect and participant as a random effect. Post hoc pairwise comparisons with a Bonferroni correction were performed to identify significant differences between specific timepoints. The findings informed our decision to combine 6‐ and 12‐month timepoints and use the maximum score from either timepoint for participants with both measurements.

Two models were created to examine predictors of sound quality ratings as measured using the SSQ‐12 Qualities scores. Specifically, pre‐activation and post‐activation models were generated, representing two clinical decision points where different intervention approaches might be considered. The pre‐activation model examined how demographic factors, pre‐op (T1) audiometric measures, and electrode placement characteristics predicted early post‐activation sound quality (T2 SSQ‐12 qualities scores), while controlling for baseline (T1) SSQ‐12 qualities scores. Including baseline scores as covariates allowed us to model change in sound quality perception rather than simply absolute scores, thus isolating factors that specifically predict improvement or decline from baseline. The post‐activation model investigated how demographic factors, early post‐activation (T2) audiometric measures, and electrode placement characteristics predicted long‐term sound quality outcomes (T3/T4 SSQ‐12 qualities scores), while controlling for early post‐activation (T2) SSQ‐12 qualities scores. We chose to focus on early post‐activation predictors rather than pre‐op factors for the long‐term model because we were interested in identifying potentially modifiable factors during the adaptation period that might influence long‐term outcomes. Model selection was performed using backward stepwise regression based on Akaike information criterion (AIC), with variables removed sequentially if their exclusion did not increase AIC by more than two points. Statistical significance was set at *p* < 0.05, and 95% confidence intervals were calculated.

## 3 Results

The study cohort consisted of 339 adult CI recipients. Participant demographic information, device characteristics, and outcome measures at each timepoint are presented in Table 1.

**TABLE 1:** Participant demographics.

Biological sex	151 Females (45%) 188 Males (55%)
Age at CI (years)	63.8 ± 15.7 [18, 92]
Onset of deafness	12 Prelingual (4%) 327 Postlingual (96%)
Ipsilateral PTA (dB HL)	Pre‐op (*N* = 335) 92.1 ± 17.3 [53, 120] 1‐month post (*N* = 174) 93.0 ± 16.3 [51,120] 6‐month post (*N* = 184) 91.9 ± 16.9 [52, 119] 12‐months post (*N* = 168) 94.3 ± 15.0 [55, 120]
Contralateral PTA (dB HL)	Pre‐op (*N* = 331) 75.1 ± 24.7 [5, 119] 1‐month post (*N* = 35) 88.7 ± 31.9 [1, 119] 6‐month post (*N* = 27) 91.3 ± 21.5 [42, 119] 12‐months post (*N* = 112) 64.8 ± 29.0 [10, 120]
CI manufacturer	77 Advanced bionics (23%) 192 Cochlear (57%) 70 MED‐EL (21%)
Electrode array type	123 Precurved (37%) 212 Straight (63%)
Scalar location	142 Scala tympani (77%) 43 Translocated to scala vestibuli (23%)
Mean modiolar distance (mm)	(*N* = 185) 0.8 ± 0.3 [0.1, 1.4]
Angular insertion depth (degrees)	(*N* = 185) 432.2 ± 91.8 [204.1, 900.3]
Extra‐cochlear electrodes (number)	(*N* = 185) 0.0 ± 0.2 [0, 1]
Aided CNC word scores (% correct)	Pre‐op (*N* = 322) 12.5 ± 15.1 [0, 86] 1‐month post (*N* = 325) 33.5 ± 22.0 [0, 86] 6‐months post (*N* = 312) 46.2 ± 22.3 [0, 92] 12‐months post (*N* = 271) 50.8 ± 22.0 [0, 90]
CI processor datalogging (hours/day)	1‐month post (*N* = 242) 10.6 ± 3.5 [0, 17.0] 6‐months post (*N* = 249) 10.6 ± 4.4 [0, 19.2] 12‐months post (*N* = 215) 11.1 ± 4.1 [0, 18.6]
SSQ‐12 qualities subjective rating scores (0–10; 0—complete disability, 10—complete ability)	Pre‐op (*N* = 339) 2.6 ± 1.8 [0, 8.5] 1‐month post (*N* = 339) 4.7 ± 2.0 [0, 10] 6‐months post (*N* = 287) 5.3 ± 1.9 [0, 9.5] 12‐months post (*N* = 212) 5.6 ± 1.9 [0.5, 9.4]

*Note*: Imaging data (scalar location, modiolar distance, angular insertion depth, and extracochlear electrodes) were available for a subset of participants (*N* = 185) as CT imaging was not performed on all patients. Missing data were handled through multiple imputation as described in Section 2.

**FIGURE 2:**
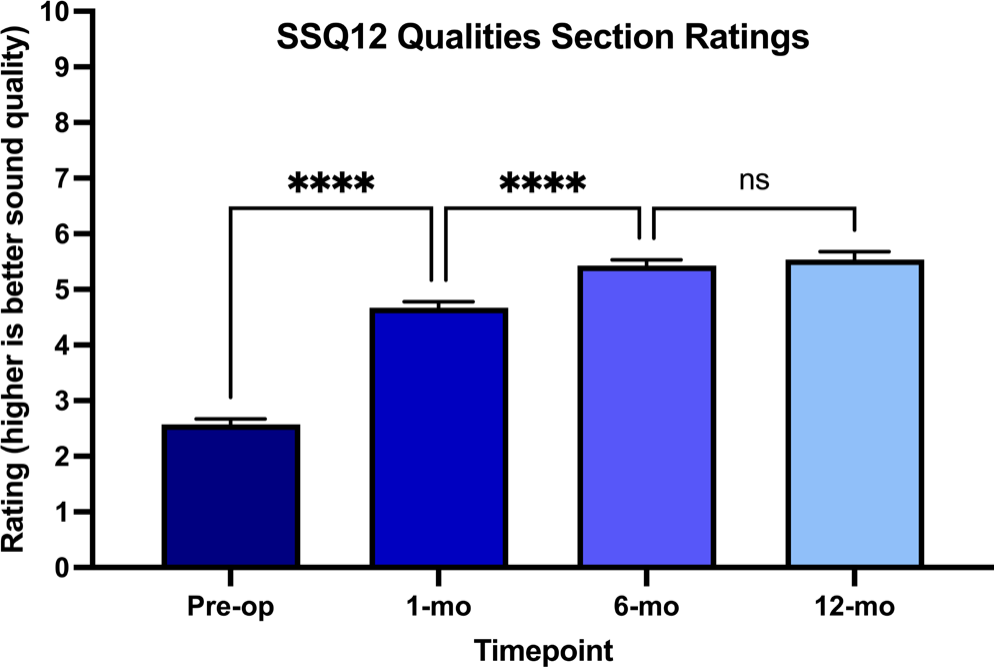
This longitudinal study of 339 adult cochlear implant recipients demonstrates that subjective sound quality ratings improve significantly during the first 6 months post‐activation before plateauing, with early outcomes predicted by electrode placement factors (closer modiolar distance and deeper insertion depth), pre‐operative sound quality, and word recognition scores. Long‐term sound quality is significantly associated with younger age, greater daily device use, and higher early post‐activation ratings. These findings highlight the importance of surgical technique and consistent device use for optimizing sound quality outcomes, while revealing the need for more sensitive assessment tools to better capture factors influencing recipients' subjective auditory experiences. [Color figure can be viewed in the online issue, which is available at www.laryngoscope.com]

**FIGURE 3:**
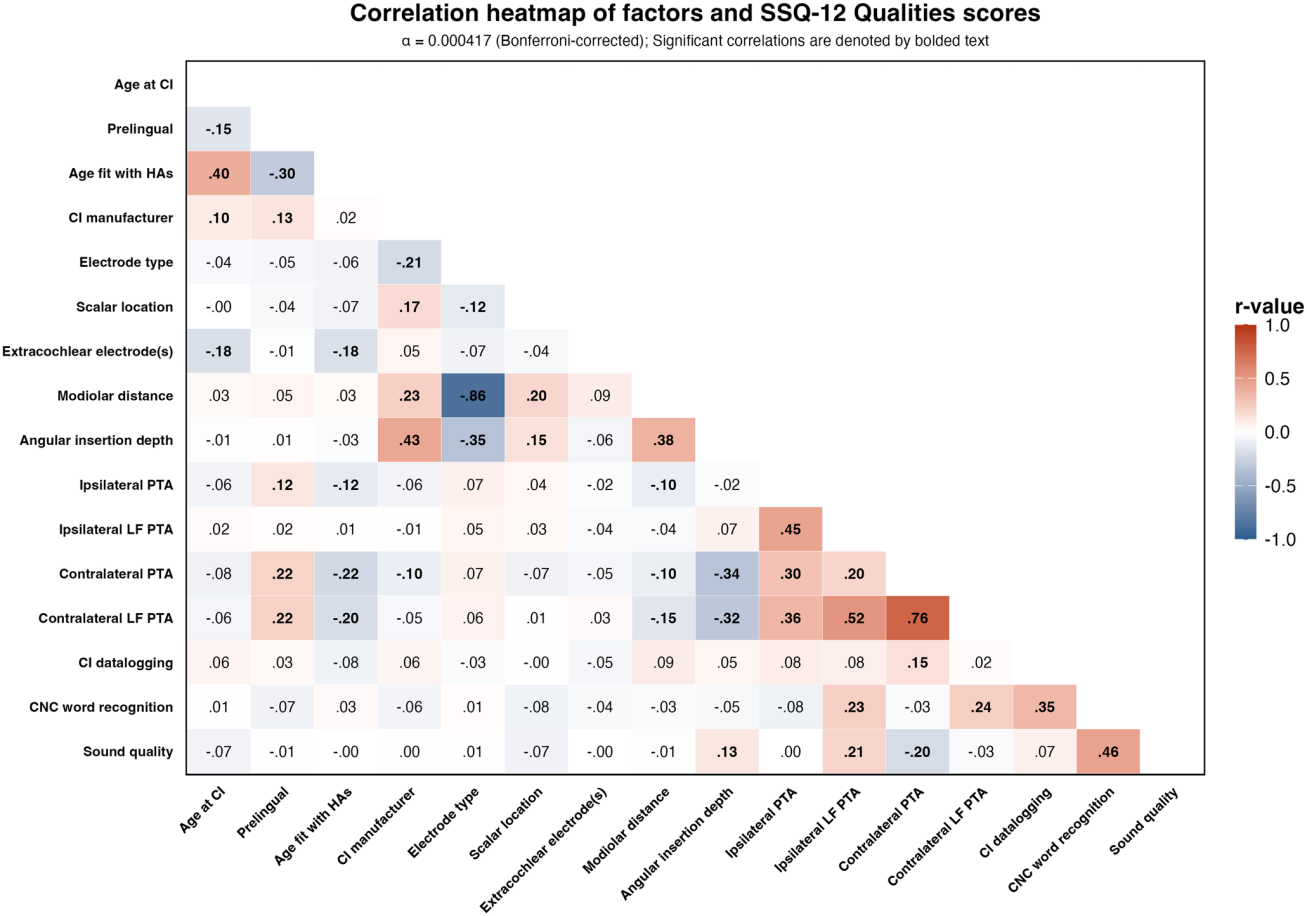
The heatmap displays correlation coefficients between all variables across timepoints, calculated using pairwise complete observations from the first imputed dataset. Color intensity and corresponding numerical values represent the strength and direction of relationships, with darker colors indicating stronger correlations. A Bonferroni correction was applied to account for multiple comparisons (α = 0.000417). Significant correlations are bolded. Abbreviations: CI, cochlear implant; HA, hearing aid; LF, low frequency; PTA, pure tone average. [Color figure can be viewed in the online issue, which is available at www.laryngoscope.com]

SSQ‐12 Qualities scores showed progressive improvement over time post‐activation (Figure 2), increasing from a mean of 2.57 (SD = 1.79, *n* = 339) pre‐operatively to 4.67 (SD = 2.00, *n* = 339) at 1‐month, 5.33 (SD = 1.92, *n* = 287) at 6‐months, and 5.64 (SD = 1.85, *n* = 212) at 12‐months post‐activation. A mixed‐effects model showed significant differences in sound quality ratings across timepoints (*F* (3, 862) = 253.76, *p* < 0.0001). Post hoc pairwise comparisons with a Bonferroni correction revealed significant differences between pre‐op and 1‐month (mean difference = 2.10, *p* < 0.0001) and between 1‐month and 6‐months (mean difference = 0.68, *p* < 0.0001), but not between 6‐months and 12‐months (mean difference = 0.20, *p* = 0.913). Given the non‐significant difference between 6‐ and 12‐months, grouping these two timepoints together seemed reasonable. A correlation heatmap (Figure 3) was constructed to examine relationships between all collected variables.

The pre‐activation model (Table 2) was significant (F (7, 331) = 10.18, *p* < 0.001) and explained 16% of the variance (adjusted *R* ^2^ = 0.16) in early (1‐month) sound quality ratings. Four factors emerged as significant predictors: better pre‐op aided CNC word scores in the ear to be implanted (estimate = 0.02, *p* < 0.001, 95% CI [0.01–0.03]), better pre‐op sound quality ratings (estimate = 0.23, *p* < 0.001, 95% CI [0.10–0.36]), closer modiolar distance (estimate = −1.03, *p* = 0.025, 95% CI [−1.91 to 0.15]), and deeper angular insertion depth (estimate = 0.004, *p* = 0.035, 95% CI [0.0002–0.007]).

**TABLE 2:** Pre‐activation predictors of early (1‐month) sound quality ratings.

	Estimate	95% CI	Std. error	*t*	*p*
(Intercept)	2.32	−0.24 to 4.88	1.31	1.77	0.084
Age	−0.001	−0.01 to 0.01	0.007	−0.19	0.853
Pre‐ versus post‐lingual	0.81	−0.39 to 2.01	0.61	1.32	0.188
Angular insertion depth	**0.004**	**0.0002** to **0.007**	**0.002**	**2.17**	**0.035**
PTA in ipsilateral ear	0.003	−0.02 to 0.02	0.009	0.36	0.723
Average modiolar distance	**−1.03**	**−1.91** to **0.15**	**0.45**	**−2.31**	**0.025**
Pre‐op CNC word score	**0.022**	**0.01–0.03**	**0.005**	**4.44**	**< 0.001**
Pre‐op SSQ‐12 qualities score	**0.23**	**0.10–0.36**	**0.06**	**3.63**	**< 0.001**

*Note*: Adjusted *R* ^2^ = 0.16, *F* (7, 331) = 10.18, *p* < 0.001, *N* = 339. Bold values statistically significance *p* < 0.000417.

The post‐activation model (Table 3) was significant (*F* (8, 330) = 22.85, *p* < 0.001), demonstrating higher explanatory power, accounting for 35% of the variance (adjusted *R* ^2^ = 0.35) in long‐term (6–12 months) SSQ qualities scores. Three predictors emerged as significant: younger age (estimate = −0.01, *p* = 0.036, 95% CI [−0.02 to 0.001]), more daily CI processor use (estimate = 0.07, *p* = 0.024, 95% CI [0.01–0.13]), and better 1‐month post‐activation SSQ‐12 qualities scores (estimate = 0.49, *p* < 0.001, 95% CI [0.40–0.58]).

**TABLE 3:** Early (1‐month) post‐activation predictors of long‐term (6–12 months) sound quality ratings.

	Estimate	95% CI	Std. error	*t*	*p*
(Intercept)	**2.84**	**1.06–4.62**	**0.91**	**3.12**	**0.002**
Age	**−0.01**	**−0.02–0.001**	**0.006**	**−2.10**	**0.036**
Pre‐ versus post‐lingual	0.14	−0.87‐1.16	0.52	0.28	0.782
Angular insertion depth	0.001	−0.002‐0.004	0.001	0.77	0.447
PTA in ipsilateral ear	−0.001	−0.01‐0.01	0.006	−0.15	0.880
CI datalogging	**0.07**	**0.01–0.13**	**0.03**	**2.29**	**0.024**
Average modiolar distance	0.002	−0.92‐0.92	0.47	0.004	0.997
1‐mo CNC word score	0.005	−0.004‐0.01	0.004	1.22	0.221
1‐mo SSQ‐12 qualities score	**0.49**	**0.40–0.58**	**0.05**	**10.17**	**< 0.001**

*Note*: Adjusted *R* ^2^ = 0.35, *F* (8, 330) = 22.85, *p* < 0.001, *N* = 339. Bold values statistically significance *p* < 0.05.

## 4 Discussion

This longitudinal study investigated the trajectory of subjective sound quality ratings and identified key predictors of early and long‐term sound quality outcomes in a large cohort of adult CI recipients. Our findings revealed several important patterns with implications for clinical practice and patient counseling.

### 4.1 Sound Quality Trajectory

In support of our hypothesis, subjective sound quality improved significantly through the first month post‐activation and stabilized between 6 and 12 months paralleling known speech recognition patterns [10, 11]. The initial improvement likely reflects the immediate benefit of restored auditory input, while gains between 1‐ and 6‐months may follow programming adjustments typically made at the 1‐month follow‐up, such as optimizing upper and lower stimulation levels using evoked stapedial reflex thresholds (eSRTs) and aided detection thresholds, respectively [11]. The plateau between 6 and 12 months suggests sound quality stabilizes earlier than previously thought [12], and should be considered for setting and managing appropriate expectations.

### 4.2 Predictors of Early Sound Quality Outcomes

The pre‐activation model identified four significant predictors of early (1‐month) sound quality ratings: modiolar distance, angular insertion depth, pre‐op sound quality ratings, and CNC scores. Pre‐op sound quality ratings likely reflect pre‐existing perceptual frameworks or hearing experiences that influence initial CI adaptation, while better pre‐op speech understanding may indicate preserved higher‐level auditory processing capabilities that facilitate faster adaptation to the CI. This aligns with previous studies, showing that pre‐op speech recognition scores predict post‐op speech recognition scores [13, 21, 22, 23].

The significant effects of electrode placement—with closer modiolar distance and deeper angular insertion both predicting better early sound quality ratings—aligned with established relationships between electrode placement and speech recognition [24, 25, 26]. However, the relative magnitude of these effects differed substantially. Modiolar distance demonstrated a larger effect size (β = −1.03) compared to insertion depth (β = 0.004), suggesting that electrode proximity to neural targets may be more beneficial for sound quality relative to a deeper insertion depth. From a clinical perspective, patients with arrays placed approximately 1.03 mm closer to the modiolus achieved meaningfully better sound quality ratings (1‐point improvement). In practical terms, this might mean selecting a precurved array rather than a straight array. In contrast, angular insertion depth required very large differences of approximately 250° to produce a similar improvement. This pattern suggests that modiolar proximity may be relatively more important than insertion depth for achieving maximal sound quality ratings, and that electrode placement plays an important role not only in speech perception, but also in subjective sound quality outcomes.

Interestingly, despite correlations between scalar location and early sound quality, scalar location was not a significant independent predictor—contrary to our hypothesis and previous literature demonstrating its importance for speech recognition [24, 25, 26]. This suggests that scalar location may contribute to sound quality perception but with a less direct influence than on speech recognition. One possibility is that scalar location effects may overlap with other variables included in the model, diluting its independent effect. Alternatively, scalar location may affect clarity in speech recognition more than subjective sound quality dimensions captured by the SSQ‐12.

### 4.3 Predictors of Long‐Term Sound Quality Outcomes

The post‐activation model identified three significant predictors for long‐term outcomes: younger age, greater daily CI processor use, and higher 1‐month post‐activation sound quality ratings. The age effect, while small, is consistent with previous work demonstrating the negative impact of advancing age on CI outcomes [13, 22, 23], likely reflecting age‐related changes in cognition, such as inhibition–concentration, working memory capacity, speed of lexical access, and non‐verbal reasoning, and extends this finding to subjective ratings of sound quality [27, 28]. Importantly, age‐related declines could impact not only auditory perception but also metacognitive appraisal of sound quality, affecting how older adults interpret and rate their experience using the SSQ‐12. This finding underscores the importance of early intervention when possible, as the average CI candidate waits 2–7 years before pursuing surgery, and it seems that older CI recipients may experience more limited sound quality improvements [29, 30]. The positive association between CI processor use and sound quality ratings supports previous findings [31], and reinforces the importance of counseling patients about the known causal link between full‐time (all waking hours) device use and improved speech recognition, to maximize long‐term outcomes [32]. Notably, early sound quality ratings strongly predicted long‐term ratings, suggesting remarkable stability in individual perceptual frameworks over time. This stability could allow clinicians to identify patients needing intervention, such as additional programming or auditory training, based on initial ratings post‐activation.

The dissociation between early and long‐term predictors was particularly intriguing. CNC word scores significantly predicted early sound quality ratings but did not emerge as a significant predictor of long‐term ratings. This pattern parallels findings demonstrating that speech recognition and quality of life outcomes are largely independent [5, 6]. Our results suggest that pre‐op speech recognition may provide an initial framework for sound quality perception that becomes less relevant over time as CI‐specific listening strategies develop. This aligns with the observations of Dorman et al., who found that CI recipients' descriptions of sound quality evolve from “computer‐like” or “metallic” initially to predominantly “clear” after extended use [12], suggesting a fundamental shift in perceptual processing that transcends pre‐implant abilities. The diminishing influence of pre‐op speech recognition abilities on long‐term sound quality ratings further supports the notion that electric hearing ultimately evolves as a distinct perceptual modality, shaped more by post‐activation experiences, as reflected by the strong predictive value of 1‐month sound quality ratings, than by pre‐op hearing abilities.

## 5 Limitations and Future Directions

A limitation revealed by this study is that previous SSQ‐12 scores consistently dominated as predictors of future sound quality outcomes, with all other factors showing modest effect sizes by comparison. Pre‐operative ratings predicted early outcomes, and early ratings strongly predicted long‐term outcomes. While this suggests remarkable stability in individual perceptual frameworks, it also indicates that the SSQ‐12 may primarily capture stable individual traits rather than being sensitive to modifiable factors such as daily processor use. The dominance of baseline scores as predictors, coupled with the modest effect sizes observed for all other significant predictors—including electrode placement (modiolar distance and angular insertion depth), age, device usage, and pre‐operative speech recognition—suggests that the SSQ‐12 qualities subscale may not adequately capture the full range of factors that influence subjective sound quality perception. This highlights an urgent need for developing more sensitive, comprehensive patient‐reported outcome measures specifically designed to assess sound quality in CI recipients.

This measurement limitation is compounded by the fact that the four items in the SSQ‐12 qualities subscale were not designed to be analyzed independently and likely do not capture all relevant domains of sound quality that are meaningful to CI recipients. The subscale focuses on clarity and listening effort, but may miss other important aspects of sound quality such as naturalness, pitch, or reverberance that could be more responsive to clinical interventions.

A further limitation stems from this study's retrospective design, which resulted in missing data from clinical visits and prevented us from controlling for potential confounding variables, such as cognitive abilities, musical experience, and specific pre‐operative counseling approaches—all of which may significantly influence subjective sound quality ratings. Despite our large sample size, missing data necessitated multiple imputation, which may have introduced bias despite our conservative statistical approach. Although multiple imputation helped preserve statistical power for variables with occasional missing values, this approach has inherent limitations as imputed values are constrained by the patterns and relationships in the observed data. Additionally, while manufacturer data were collected, we excluded this variable from our final models due to multicollinearity with electrode design characteristics, and manufacturer‐specific subgroup analyses would yield insufficient sample sizes for some device combinations.

Future work should focus on developing a more comprehensive patient‐reported outcome measure of sound quality that is specifically designed to assess the various dimensions of sound quality that recipients deem important, such as the clarity, naturalness, resonance, and distortion of particular sounds in everyday environments (e.g., kitchen, outdoors, car, office, music concert). Such measures should be designed with sufficient sensitivity to detect electrode placement effects and other surgical factors that are known to impact auditory outcomes. The development of more sensitive sound quality instruments could potentially reveal stronger relationships between electrode placement and subjective auditory experience, providing better tools for surgical planning and patient counseling.

Future research should also investigate which CI programming parameters impact sound quality perception. While our study examined demographic, audiometric, and electrode placement characteristics, we were unable to investigate the impact of specific mapping parameters, such as dynamic range, channel stimulation rate, pulse duration, frequency allocation, and signal processing strategy on sound quality ratings. Gaining a better understanding of these relationships could lead to programming interventions to maximize sound quality, rather than focusing exclusively on speech recognition. Additionally, controlled studies examining the impact of targeted auditory training on sound quality perception are needed, especially interventions applied during the first 6 months post‐activation when our data suggest the most substantial adaptation occurs. Such training might facilitate more rapid adaptation to sound quality and potentially improve long‐term outcomes for patients who perceive and express poor initial sound quality ratings.

## 6 Conclusion

This longitudinal analysis demonstrates that subjective sound quality ratings in adult CI recipients rapidly improve in the first 6 months post‐activation and plateau thereafter. While electrode placement and better pre‐op word recognition significantly predict early sound quality outcomes, younger age, greater daily CI processor use, and early post‐activation quality ratings predict long‐term sound quality ratings. These findings highlight the multifactorial nature of sound quality and point to both the significance of those early months after activation for helping patients adjust and the real benefits of consistent device use in adapting to the electrical signal. Enhancing sound quality represents an important line of CI research, as it strongly predicts CI‐related quality of life and may ultimately improve patient satisfaction, device use, and more broadly CI acceptance among individuals who could benefit from this technology.

## Conflicts of Interest

A.C.M. serves as Chief Medical Officer and on the Board of Directors for Otologic Technologies, and is a paid consultant for Amgen. K.A.B. and T.N.T. have no conflicts to disclose.

## Data Availability

The data that support the findings of this study are available from the corresponding author upon reasonable request.
